# Antibodies against measles and rubella virus among different age groups in Thailand: A population-based serological survey

**DOI:** 10.1371/journal.pone.0225606

**Published:** 2019-11-26

**Authors:** Nasamon Wanlapakorn, Rujipat Wasitthankasem, Preeyaporn Vichaiwattana, Chompoonut Auphimai, Pornsak Yoocharoen, Sompong Vongpunsawad, Yong Poovorawan

**Affiliations:** 1 Center of Excellence in Clinical Virology, Department of Pediatrics, Faculty of Medicine, Chulalongkorn University, Bangkok, Thailand; 2 Division of Academic Affairs, Faculty of Medicine, Chulalongkorn University, Bangkok, Thailand; 3 National Biobank of Thailand, National Science and Technology Development Agency, Pathum Thani, Thailand; 4 Department of Disease Control, Division of Vaccine Preventable Diseases, Ministry of Public Health, Nonthaburi, Thailand; Faculty of Science, Ain Shams University (ASU), EGYPT

## Abstract

Measles and rubella are highly contagious viral diseases transmitted via respiratory secretions and aerosolized droplets. Thailand has implemented universal vaccination against measles using the monovalent measles (M) or the trivalent measles-mumps-rubella (MMR) vaccine for the past 30 years. Nevertheless, incidence of measles and rubella remains in some parts of the country. We conducted a seroprevalence study to evaluate the antibodies to measles and rubella virus among Thais of all ages and to determine pre-existing immunity resulting from either vaccination and/or natural exposure. A total of 1,781 serum samples collected in 2014 was tested for IgG to measles and rubella virus by commercial enzyme-linked immunosorbent assays (ELISA). Percentages of individuals with protective antibody levels and the geometric mean concentrations (GMC) of IgG in each age group were analysed. The GMC of anti-measles IgG and anti-rubella IgG were 653.7 IU/L (95% confidence interval (CI); 555.9–751.4) and 39.5 IU/mL (95% CI;35.0–43.9), respectively. Thais between the ages of six months and 25 years did not demonstrate sufficient protective herd immunity for measles. This observation is consistent with the recent measles outbreaks in this age group. Lower prevalence of immunity against rubella was found among children ages 5–6 years who may not have completed vaccination as infants. Our findings identify gaps in rubella and measles immunity in specific age groups and support recommendations for catch-up MMR vaccination in individuals 30 years of age or younger.

## Introduction

Measles is a highly contagious viral disease which has also been associated with complications including pneumonia, myocarditis, encephalitis, and subacute sclerosing panencephalitis. It is one of the leading causes of death among young children worldwide, with a fatality rate as high as 15% in children [1]. Even though a safe and cost-effective vaccine is available, there were 110,000 measles deaths globally in 2017, mostly among children under age 5 years [2]. In 2012, the World Health Organization released a Global Vaccine Action Plan aiming to eliminate measles and rubella by the year 2020. This goal of elimination requires a continued commitment to increase vaccination coverage levels and to conduct seroepidemiological surveys, to establish the population at risk of contracting the infection.

In Thailand, in the pre-vaccination era, the incidence of measles ranged from 70 to 90 cases per 100,000 population per year, with the highest incidence found in children younger than 5 years of age [3]. In 1984, a first dose of measles vaccine was incorporated into the national immunization program for Thai children in the form of monovalent measles (M) vaccine given to infants at 9–12 months of age. In 1996, the second dose of measles vaccine, which was replaced by trivalent measles–mumps–rubella (MMR) vaccine in 1997, was administered to 6-year-old children. This was a school-based program that offered the vaccine to first-grade students. The incidence of measles decreased dramatically after this two-dose universal vaccination program but still ranged between 1.5 and 8 cases per 100,000 population [3]. The peak incidence for severe measles was found in children under 5 years of age. Since 2014, the Expanded Program on Immunization (EPI) in Thailand has changed the second-dose measles vaccination to age 2.5 years, to induce protective immunity in children who may have had primary vaccine failure owing to maternal immunity (Table 1).

**Table 1:** Measles vaccination in the Expanded Program on Immunization (EPI) of Thailand.

Year	Primary immunization	Booster	
	9–12 months	6 years	2.5 years
1984–1996	M	−	−
1996–1997	M	M	−
1997–2010	M	MMR	−
2010–2014	MMR	MMR	−
2014-present	MMR	−	MMR

M; Measles vaccine, MMR; Measles-mumps-rubella vaccine.

Rubella is a self-limitingviral illness; however, infection during the first trimester of pregnancy can result in a severe illness of infants called congenital rubella syndrome (CRS). In the pre-vaccination era, the number of reported cases of rubella in Thailand was 17.50 per 100,000 people, with the highest incidence found among children and young adults from 5 to 24 years old [4]. Rubella (R) immunization was first given to female sixth-grade students (aged 11–12) years during 1986–1993 to prevent CRS, and later, to 6-year-old students of both sexes during 1993–1997, before being replaced by the MMR vaccine in 1997. In 2010, Thailand started two-dose rubella immunization by replacing the M with the MMR vaccine in infants aged 9–12 months and continuing the second MMR dose at 6 years of age. In 2014, the timing of the second dose of rubella vaccine was changed to age 2.5 years to boost immunity against measles, as mentioned above (Table 2). Since rubella vaccine implementation, the incidence of rubella in Thailand has remained low, with a reported rubella morbidity rate in 2003–2017 of less than one case per 100,000 people. This incidence could be underestimated as there were limited laboratory facilities available to confirm the diagnosis of rubella in patients presenting with fever and rash.

**Table 2:** Rubella vaccination in the Expanded Program on Immunization (EPI) of Thailand.

Year	Primary immunization			Booster	
	9–12 months	6 years	12 years (girls only)	6 years	2.5 years
1986–1993	−	−	R	−	−
1993–1997	−	R	−	−	−
1997–2010	−	MMR	−	−	−
2010–2014	MMR	−	−	MMR	−
2014-present	MMR	−	−	−	MMR

R; Rubella vaccine, MMR; Measles-mumps-rubella vaccine.

Despite the high vaccine coverage resulting in decreased disease prevalence, measles is still detected in several parts of Thailand, especially in the south [3]. In this study, we conductedmeasles and rubella serological surveillance in the general population from four geographical regions of Thailand, to identify susceptible individuals and provide evidence-based guidelines for the national vaccine committee regarding a catch-up campaign.

## Material and methods

### Study population and survey design

This study was conducted according to the Declaration of Helsinki and Good Clinical Practice (ICH-GCP) guidelines and approved by the Institutional Review Board of the Faculty of Medicine, Chulalongkorn University (IRB No. 259/61). We investigated measles and rubella immunity using stored serum samples that were collected between April and October 2014 for evaluation of the impact of the universal hepatitis B immunization program [5]. The seroprevalence of immunoglobulin G (IgG) antibodies to mumps has been evaluated and published previously [6]. We randomly selected serum samples from people aged between 6 months and 71 years from four provinces representing northern (n = 459), northeastern (n = 477), central (450), and southern (n = 395) Thailand. Participants were healthy children attending scheduled pediatric health check-ups or people visiting outpatient clinics at provincial hospitals. The inclusion and exclusion criteria used in this study have been published previously [5]. As vaccine records were unavailable, we assumed that individuals had received measles and rubella vaccination, according to the EPI of Thailand. Serum samples were stored at −70°C until testing.

### Laboratory testing

Commercial enzyme-linked immunosorbent assay (ELISA) kits were used to determine the concentration of IgG against measles and rubella (EUROIMMUN, Lübeck, Germany). Serum samples were initially diluted 1:101, and further dilutions were performed to yield results within the detection range, according to the manufacturer’s instructions. The concentration values for anti-measles IgG and anti-rubella IgG were expressed in international units per liter (IU/L) and IU per milliliter (IU/mL), respectively. The internationally assigned protective levels of immunity for discriminating between susceptible and protected individuals are ≥ 200 IU/L [7] and ≥ 10 IU/mL [8] for measles and rubella, respectively. Antibody against measles and rubella were divided into five groups, according to immunity levels (measles: < 200 IU/L, 200 to <275 IU/L, 275 to <1000 IU/L, 1000 to <5000 IU/L and ≥5000 IU/L, and rubella: < 8 IU/mL, 8 to <11 IU/mL, 11 to <50 IU/mL, 50 to <200 IU/mL and ≥200 IU/mL). Antibody levels below the lower limit of quantification (LLOQ) observed in some samples were assumed to be equivalent to half the LLOQ (25 IU/L for anti-measles IgG and 0.5 IU/mL for anti-rubella IgG).

### Description of notification system and data source

Thailand has implemented measles surveillance since 1980 and rubella surveillance since 1984. Annual incidence rates were measured per 100,000 inhabitants, stratified by age group, as follows: < 28 days;>28 days to 1 year; and 1, 2, 3, 4, 5, 6, 7–9, 10–14, 15–24, 25–34, 35–44, 45–54, 55–64 and >65 years old. The clinical criteria of measles were defined as presence of fever over 38°C and erythematous rash and cough in association with other symptom(s) as follows: coryza, conjunctivitis, and Koplik spots. A suspected case was defined as a person with clinical symptoms or diagnosed by a medical professional. Physiciansare encouraged to report all suspected measles cases and, whenever possible, specimens should be collected and processed for laboratory confirmation [9]. Nevertheless, less than half of suspected cases are confirmed by a laboratory. In rubella surveillance, the case definition includes low-grade fever and erythematous rash in association with one of the following symptoms: generalized lymphadenopathy, arthralgia, arthritis, and conjunctivitis [4]. The reporting criteria includes suspected cases. If CRS is suspected, prompt reporting to the health center responsible for epidemiological investigation is necessary, to confirm the diagnosis. In this study, the incidence of measles and rubella in the different age groups between 2011 and 2018 was also analyzed, together with the immunological data.

### Data and statistical analysis

Sample sizes were calculated based on the seroprotection rate (SPR) against measles described in serological surveillance data of 2004 [10]. A total 1,781 samples from individuals across all age groups were randomly selected from among 5,964 samples. Age groups were re-categorized, according to acquisition of protective immunity. IgG levels were expressed as the geometric mean concentration (GMC) with 95% confidence interval (CI). Differences in the SPR between strata of agegroup and sex were evaluated using the chi-square test. A *p*-value of < 0.05 was considered statistically significant.

## Results

### Anti-measles IgG

The 1,781 serum samples were divided into six age groups (0–1, 2–6, 7–14, 15–25, 26–30, and > 30 years), based on acquisition of anti-measles IgG (Table 3). The overall GMC of anti-measles IgG in the population was 653.7 IU/L (95% CI: 555.9–751.4). The highest GMC was found in samples from individuals aged > 30 years (GMC 1,242.0 IU/L, 95% CI: 1,002.3–1,481.7), who were born before the EPI began. The lowest GMC was found in the age group 7–14 years (GMC 360.4 IU/L, 95% CI: 257.1–463.8) (Table 4).

**Table 3:** Age group classification based on acquisition of anti-measles and anti-rubella IgG.

Mode of acquisition	Age as of 2014 (years)	Birth year
**Anti-measles IgG immunity**		
Maternal immunity orpost 1^st^ dose vaccination	**0–1**	2013–2014
Post 1^st^ dose vaccination	**2–6**	2008–2012
Post 2^nd^ dose vaccination	**7–14**	2000–2007
Post 2^nd^ dose vaccination	**15–25**	1989–1999
Post single-dose vaccination (coverage 6–70%)	**26–30**	1984–1988
Natural immunity	**>30**	Before 1984
**Anti-rubella IgG immunity**		
Maternal immunity orpost 1^st^ dose vaccination	**0–1**	2013–2014
Post 1^st^ dose vaccination at 9-12-month-old	**2–4**	2010–2012
No vaccination at all	**5–6**	2009–2010
Post single-dose vaccination at 7-year-old	**7–28**	1986–2008
Female vaccination at 12 years/ male natural immunity	**29–40**	1974–1985
Natural immunity	**>40**	Before 1974

**Table 4:** Geometric mean concentrations (GMC) of anti-measles and anti-rubella IgG.

**Age groups (years)**	**Sample size**	**GMC (IU/L)**	**95% CI**	**SPR (%)**	**95% CI**
**Anti-measles IgG**					
**0–1**	161	522.4	(284.6,760.2)	71.4	(64.4,78.4)
**2–6**	336	505.9	(397.9,613.8)	80.7	(76.5,84.9)
**7–14**	270	360.4	(257.1,463.8)	74.1	(68.9,79.3)
**15–25**	306	392.4	(248.5,536.3)	73.2	(68.2,78.2)
**26–30**	104	976.8	(653.1,1300.4)	92.3	(87.2,97.4)
**>30**	604	1242.0	(1002.3,1481.7)	98.7	(97.8,99.6)
**Total**	1781	653.7	(555.9,751.4)	84.3	(82.6,86.0)
**Age groups (years)**	**Sample size**	**GMC (IU/mL)**	**95% CI**	**SPR (%)**	**95% CI**
**Anti-rubella IgG**					
**0–1**	161	63.7	(46.6,80.8)	72.0	(65.1,78.9)
**2–4**	219	41.0	(32.0,49.9)	79.5	(74.2,84.8)
**5–6**	117	17.8	(0.9,34.8)	29.1	(20.9,37.3)
**7–28**	635	32.3	(26.8,37.9)	83.9	(81.0,86.8)
**29–40**	244	43.1	(30.9,55.3)	87.3	(83.1,91.5)
**>40**	405	46.5	(34.9,58.1)	81.0	(77.2,84.8)
**Total**	1781	39.5	(35.0,43.9)	78.5	(76.6,80.4)

CI; Confidence Interval, SPR, seroprotective rate. The unit for anti-measles and anti-rubella IgG was IU/L and IU/mL, respectively

**Fig 1:**
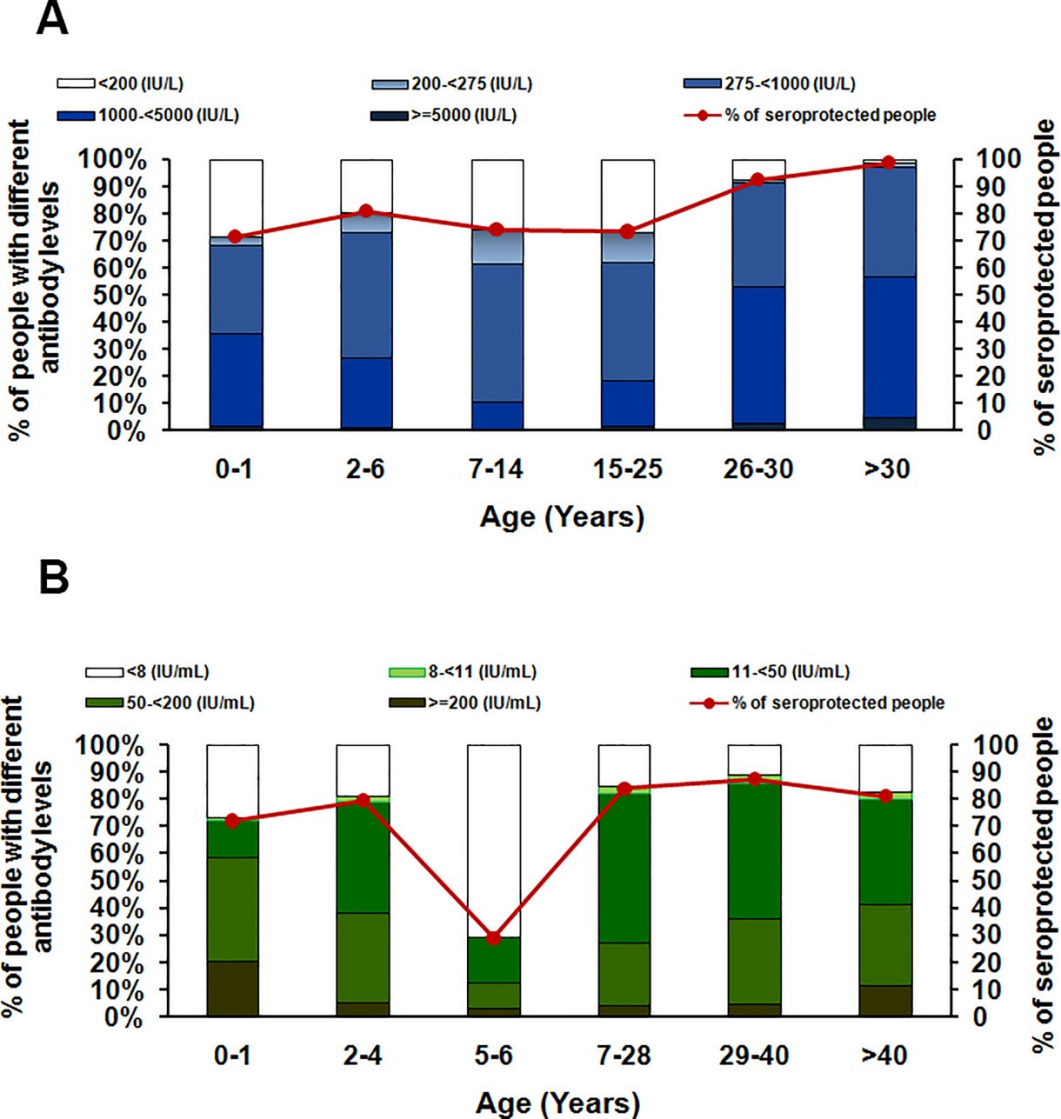
**Age-specific A) anti-measles and B) anti-rubella IgG from study participants across all age groups**. Age groups were re-categorized according to acquisition of protective immunity. Scale on the left represented the percentages of population with different antibody levels. Scale on the right represented the percentages of seroprotected individuals according to the cut-off levels of measles IgG ≥ 200 IU/L and rubella IgG ≥ 10 IU/mL.

When samples were classified according to antibody levels (Fig 1A), we found that most of the population had antibody levels 275–1000 IU/L (42.8%), followed by 1000–5000 IU/L (32.8%). The overall percentage of individuals protected against measles in the population was 84.3% (95% CI: 82.6–86.0). The highest seroprotection rate (levels ≥200 IU/L) was observed in the age group > 30 years who were naturally infected (98.7%), followed by the groups aged 26–30 years (92.3%) and 2–6 years (80.7%). The results showed that SPRs in people born before the EPI program (aged > 30 years) were significantly higher than in those born after the vaccination period (age ≤ 30 years) (98.7% vs. 75.5%, p < 0.001). There were no significant differences in the SPR between male and female populations. However, a comparison of the measles SPR by residence among the four provinces showed that residents of Khon Khan had a lower SPR(80.3%) than residents of other provinces (Ayuthaya: 84.0%, Uttradit: 86.7%, and Narathiwat: 86.8%). We found that even though measles immunization has been implemented for more than 30 years, Thai individuals between the ages of 6 months and 25 years did not demonstrate sufficient herd immunity protection against measles, which is 90%–95% [11]. This observation is consistent with the recent measles outbreaks among children and young adults below age 30 years (S1 Fig).

### Anti-rubella IgG

The 1,781 samples were divided into six age groups (0–1, 2–4, 5–6, 7–28, 29–40, and >40 years), based on acquisition of anti-rubella IgG (Table 1). Because rubella vaccine in infants was recently implemented in 2010, children born between 2009 and 2010, aged between 5 and 6 years at the time of survey in 2014, may have missed the first dose as infants and they were not yet old enough to receive the second dose at age 6 years. This corresponds well with the highest seronegativity rate (levels < 8 IU/mL) of 70.9%observed in the age group 5–6 years (Fig 1B). The overall GMC of anti-rubella IgG in the population was 39.5 IU/mL (95%CI: 35.0–43.9). The highest GMC was found in individuals aged> 40 years (GMC 46.5 IU/mL, 95% CI: 34.9–58.1), who were born before the EPI began. The lowest GMC was found in the age group 5–6 years, corresponding to those missing of the first-dose rubella vaccination (Table 2).

The overall percentage of individuals protected against rubella in the population was 78.5% (95%CI: 76.6–80.4). The highest SPR was found in the age group 29–40 years, comprising females vaccinated at age 12 years and males with natural immunity (87.3%, 95%CI: 83.1–91.5). Between 1986 and 1993, rubella vaccine was only administered to girls. We compared the SPRs between male and female individuals in this age group (29–40 years old) and found that SPRs in males were significant lower than those in female individuals (78.7% vs. 90.2%, *p* = 0.023). A comparison of the rubella SPR by residence in the four provinces showed that residents of Khon Khan and Ayuthaya had significantly lower seropositivity rates (76.1% and 77.1%, respectively) than residents of Uttradit (80.6%) and Narathiwat (80.5%) provinces.

The herd immunity threshold of rubella has been estimated at 85%–88% [12]. In this study, we found that Thai individuals aged < 28 years had not achieved the heard immunity targets, which corresponds to the incidence of rubella in recent years that peaked in adults under 35 years old (S2 Fig). Peak incidence was not detected in children 5–6 years of age.

## Discussion

Although Thailand has implemented two-dose measles vaccination program for more than 18 years, evidence from our study demonstrated that the threshold of herd immunity against measles has not been achieved among people under 30 years of age. Between 2004–2017, the estimated vaccination coverage in Thailand has reached >95% for the first dose and >90% for the second dose of measles-containing vaccine [13]. However, the overall SPR for measles in this study was lower than expected (84.3%), but similar to that of the 2004 survey (81%) [10]. People between the ages of 7–14 and 15–25 years, who should have received two doses of measles vaccine, had unexpectedly low SPRs for measles of 74.1% and 73.2%, respectively. This finding was similar to the 2004 survey [10]. Plausible explanations for this include the rapid waning of immunity or potency problems of the measles vaccine administered in this program. The discrepancy between the vaccine coverage and SPR has also been observed in Vietnam [14] with seroprevalence between 40% and 80% for children younger than age 5 years. The precise causes of this discrepancy warrant further investigations.

Comparisons between the SPRs of people residing in the four regions of Thailand showed that despite the high incidence of reported measles in the south [15], the SPR in Narathiwat province was unexpectedly higher than that in other regions. This may be due to the small sample size. In addition, one representative province is probably not indicative of the situation in other provinces within the region and minority populations living in particular locations.

Comparison of anti-measles seroprevalence in population across all ages between eastern and western countries showed that eastern population residing in Thailand (this study), Vietnam [14] and China [16] had lower seroprotection rate of 84–86% than western population inthe Netherlands [17] and Czech [18] (SPR 93–96%). Based on the annual report to the Ministry of Public Health, Thailand, the incidence of measles is still on the rise, especially among people less than 35 years of age [15], therefore we recommend a catch-up vaccination program for these age groups, to prevent further cases.

Because the greatest concern from a public health standpoint is preventing CRS, selective vaccination against rubella was initiated in 11-year-old girls in 1986, and this vaccination program continued until 1993. The vaccinated female population reached their 30s and 40s in 2014. As previously expected, anti-rubella IgG levels among females in this age group were significantly higher than those in their male counterparts. This gender-specific differences were also found in other studies with selective vaccination program [19–20]. Two-dose rubella immunization for infants aged 9–12 months and 6 years started in 2010. At the time of this survey in 2014, children aged 4 years and below should have received one dose during infancy. Children aged 6 years and above should have received one dose at 6 years old, thus leaving a gap for children 5–6 years old who may have missed the rubella vaccination as infants. Our results support the national schedule, with evidence of extremely low SPRs for rubella (29.1%) among this age group. In view of the age-specific differences in antibody levels and incidences, based on prevalent cases reported to the surveillance system, there were no correlations between the rubella SPR and reported incidences. Possible explanations may include underestimation of the rubella incidence in certain populations due to mild illness indistinguishable from other viral infections. The results of the one-dose rubella vaccination program in Thailand showed that certain groups between age 7 and 28 years are at risk of contracting rubella. Thus, a catch-up dose of rubella vaccine in these age groups may be necessary. However, we estimate that young children who were born under the two-dose rubella vaccination program will have higher SPRs in adulthood.

After administration of the second dose of the MMR vaccine was changed from age 6 years to 2.5 years in 2014, the vaccine coverage was unexpectedly low, according to the 2018 selectedregion vaccine coverage survey which is 95%–99% for the first dose and 78%–96% for the second dose of measles-containing vaccine [21]. The coverage was extremely low in Pattani Province, the randomly-selected site for vaccine coverage survey in the southern part of Thailand (71.3% for the first dose and 66.7% for the second dose of measles-containing vaccine) [21]. Although the vaccine coverage survey was performed in selected regions and not nation-wide, the low vaccine coverage in recent years corresponds with the rise in measles incidence between 2017 and 2018corresponding to 2981 and 5637 reported case per year, respectively [15]. Authorities working on maintaining high vaccine coverage have a crucial role in closing this gap.

Although we included a relatively large sample size, this study had limitations. One representative province in each region may not reflect the situation of that region. Besides, vaccine coverage survey was performed in different representative provinces which may result in discordance between the vaccine coverage and seroprotection rate.

The serosurveillance study afforded the opportunity to clarify the immune status of the Thai population at a given time point.These results are generalizable to the countries where the EPI is resembled or similar to Thailand, and where outbreaks of measles are ongoing or the incidence cases are rising. Susceptible groups were identified such that preventive measures (e.g., adapting vaccination strategies) can be taken to prevent further cases arising. Our findings identified gaps in rubella and measles immunity for specific age groups in the Thai population. The present results support recommendations for catch-up measles or MMR vaccination in individuals age 30 years or younger, to achieve herd immunity and prevent outbreaks of these diseases in the community.

## Supporting information

S1 Fig Numbers of reported measles cases per year by age groups in Thailand between 2011 and 2018. Data were retrieved from the official website of the Bureau of Epidemiology, Department of Disease Control, Ministry of Public Health, Thailand.
(TIF)

S2 Fig Numbers of reported rubella cases per year by age groups in Thailand between 2011 and 2018. Data were retrieved from the official website of the Bureau of Epidemiology, Department of Disease Control, Ministry of Public Health, Thailand.
(TIF)
