# Supplementary figures and images for: Antibodies against measles and rubella virus among different age groups in Thailand: A population-based serological survey
Source: PLoS One. 2019 Nov 26;14(11):e0225606. doi: 10.1371/journal.pone.0225606 (PMC6879141; doi:10.1371/journal.pone.0225606)

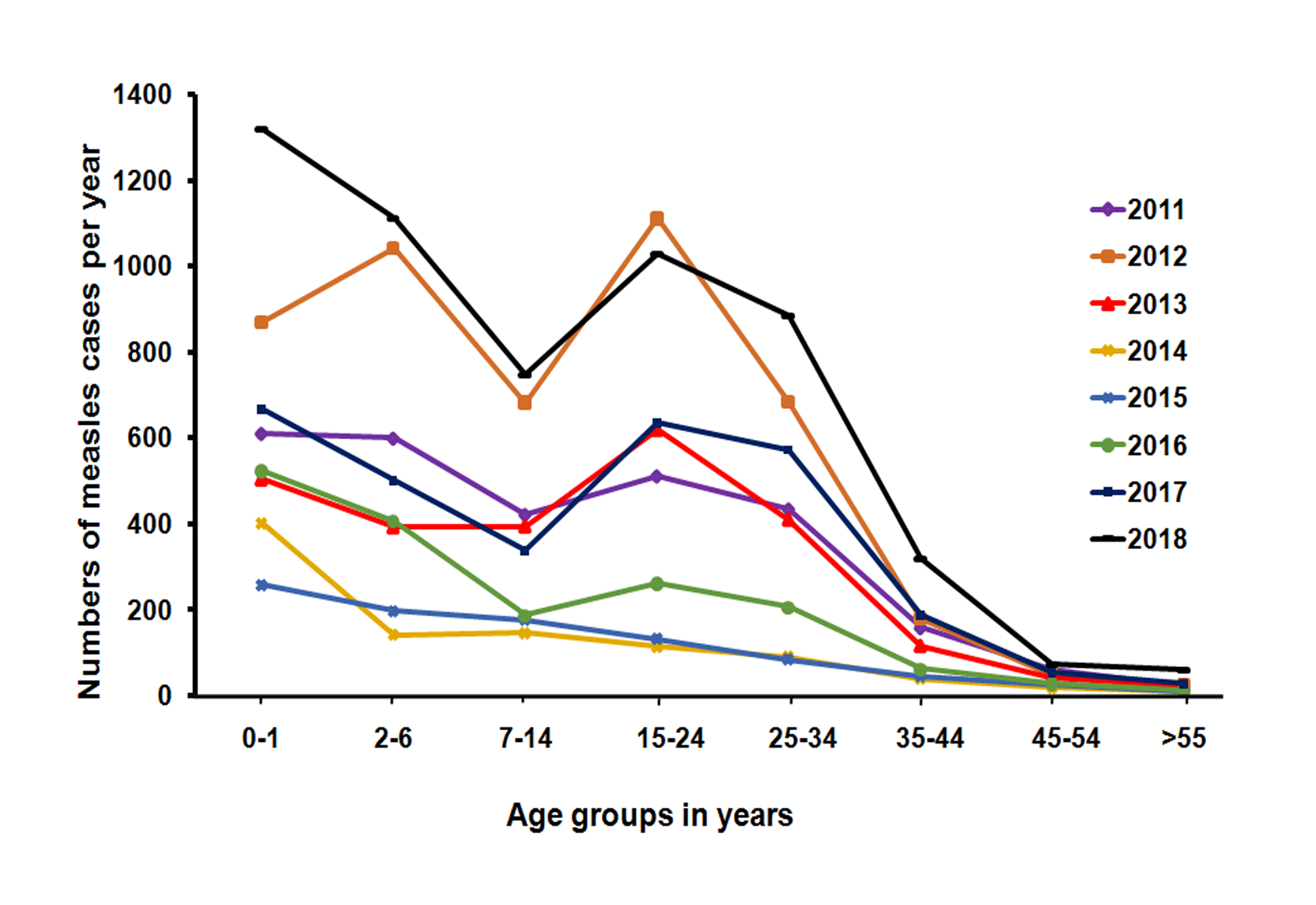

Supplement: S1 Fig — Data were retrieved from the official website of the Bureau of Epidemiology, Department of Disease Control, Ministry of Public Health, Thailand. (TIF) [file pone.0225606.s001.tif]

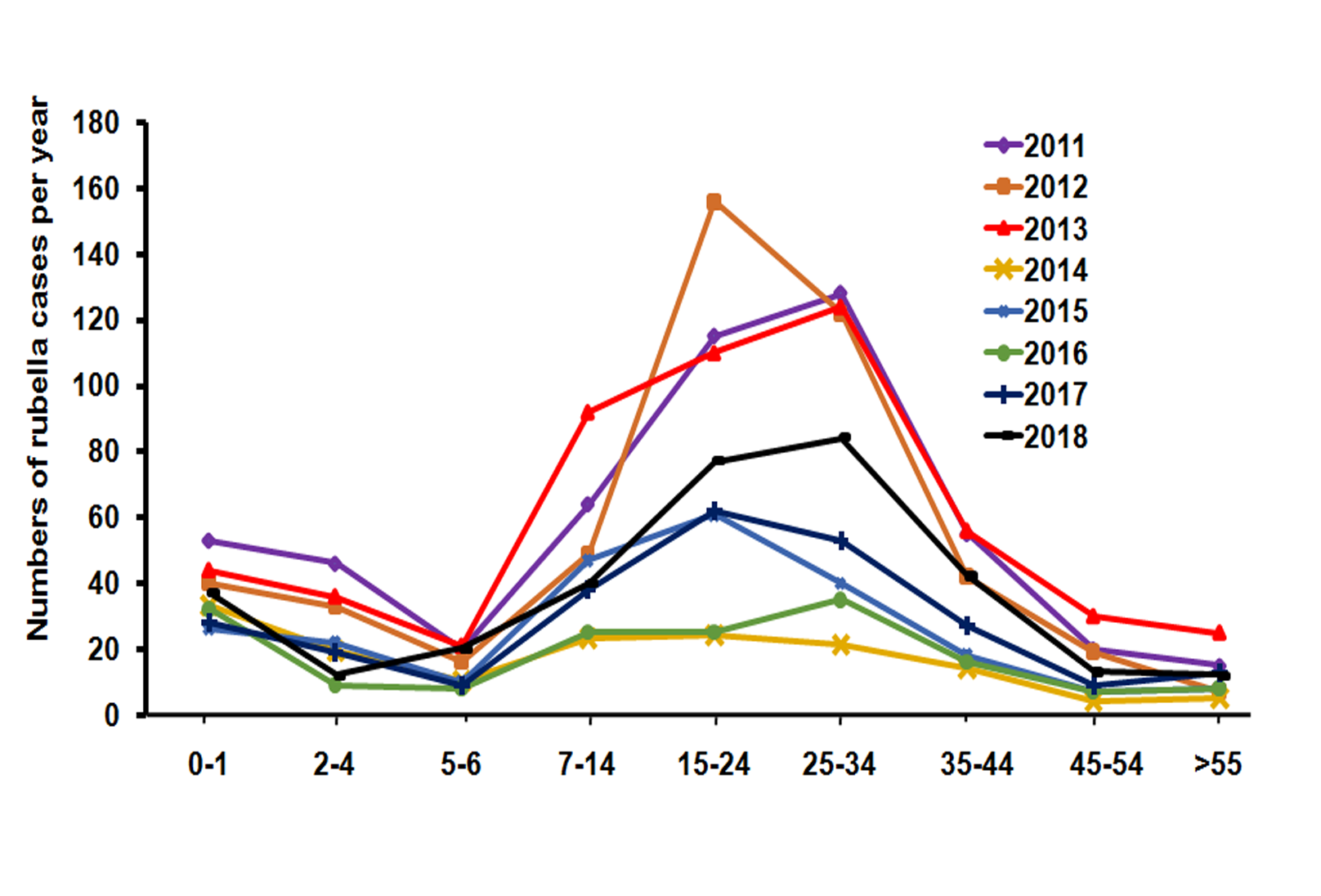

Supplement: S2 Fig — Data were retrieved from the official website of the Bureau of Epidemiology, Department of Disease Control, Ministry of Public Health, Thailand. (TIF) [file pone.0225606.s002.tif]
